# PM and Elevated Inflammation Markers: More Support for Air Pollution–Heart Disease Link

**Published:** 2007-07

**Authors:** Bob Weinhold

After evaluating about 1,000 Europeans already suffering from cardiovascular disease, researchers have found a relationship between elevated concentrations of airborne particulates (PM) and increases in two markers of inflammation that have strong links with cardiovascular diseases and deaths **[*EHP* 115:1072–1080; Rückerl et al.]**. The cumulative number of people studied is the largest to date for PM and these inflammation indicators, with the findings generally consistent across diverse locales.

The researchers evaluated about 100 to 200 myocardial infarction survivors in each of six cities around the continent: Athens, Augsburg, Barcelona, Helsinki, Rome, and Stockholm. The cities have a wide range of demographic profiles, climates, and air pollutant concentrations. The selected people tended to be male, elderly, overweight, and consumers of numerous prescription drugs.

To evaluate inflammation, the researchers studied interleukin 6 (IL-6; thought to play a central role in triggering inflammation) and two proteins, fibrinogen and C-reactive protein (CRP), whose synthesis is stimulated by IL-6. They also considered the potentially confounding effects of many other variables, such as smoking status, presence of diabetes, time of day, and season.

They found that IL-6 increased most when particle number concentration—an indicator of ultrafine particulates—was elevated 12 to 17 hours before a blood draw. They also found that increased fibrinogen was associated with cumulative five-day exposure to larger particulates (PM_10_). In addition, the results indicated associations between fine particulates (PM_2.5_) and fibrinogen, and between nitrogen dioxide and IL-6.

There were a few anomalies that remain to be explained, such as the fact that the strongest link between PM_10_ exposures and increased fibrinogen after three days occurred in Helsinki, even though that city had the lowest PM_10_ concentrations of the six studied. There were no consistent patterns for CRP, although the results may have been skewed by the fact that most of the people studied consumed statins, cholesterol-lowering drugs known to reduce this protein.

Much remains unknown about the links between the inflammation indicators tracked and subsequent health effects. Nonetheless, this study may help explain conflicting results of similar work, since it better addresses previous limitations such as lack of geographic diversity, small subject population, limited number of inflammation indicators tested, and variable health status.

